# Case Report: Management of a case of multidrug-resistant *Klebsiella pneumoniae* infection in a second-kidney transplant patient

**DOI:** 10.3389/frtra.2024.1494016

**Published:** 2025-01-17

**Authors:** Supreeta R. Shettar, Mahadevaiah Neelambike Sumana, Manjunath S. Shetty, Yogeesh D. Maheshwarappa, Reddy G. Raghukanth, Asha Srinivasan, Dharan P. Vamshi, Gautam Kalyatanda, Swamy G. S. Veerabhadra, Shylaja Eshwarappa Chinchana

**Affiliations:** ^1^ JSS Medical College and Hospital, JSS Academy of Higher Education and Research, Mysuru, India; ^2^ Department of Nanoscience and Technology, School of Life Sciences, JSS Academy of Higher Education and Research, Mysuru, India; ^3^ Division of Infectious Disease and Global Medicine, University of Florida, Gainesville, FL, United States

**Keywords:** multidrug-resistant organisms, multidrug-resistant *Klebsiella pneumoniae*, urinary tract infection, kidney transplantation, immunocompromised condition, extended *β*-lactam infusion

## Abstract

This case report on recurrent urinary tract infections (UTIs) caused by multidrug-resistant (MDR) *Klebsiella pneumoniae* in a post-renal transplant patient underscores the significant clinical challenge of managing MDR infections in immunocompromised individuals, particularly in the context of renal transplantation. The patient was treated with an extended infusion of meropenem, which offers prolonged drug exposure and enhances bactericidal activity against MDR pathogens. This approach is critical in overcoming the resistance mechanisms inherent to *Klebsiella pneumoniae*, thereby improving the likelihood of therapeutic success. The findings presented here highlight the potential efficacy of extended meropenem infusion in treating MDR infections, providing a valuable therapeutic option for clinicians facing similar cases. This report contributes to the growing evidence supporting advanced antibiotic administration techniques in managing complicated urinary tract infections in transplant in resource limited countries.

## Introduction

Infections by multidrug-resistant (MDR) bacteria have become a worldwide public health problem in recent years (1). The causes of the development and spread of these bacteria are multifactorial, based on the indiscriminate use of antimicrobials for prolonged periods, as well as through contact with contaminated healthcare workers (2). Infection by MDR bacteria generates a great impact, especially in patients subjected to solid organ transplantation (Tx) (3).

Nowadays, solid organ transplant (Tx) is considered an important strategy to treat many end-stage organ failures (3). India sees 17,000–18,000 solid organ transplants performed every year, the most in the world after the United States and China but remains behind several high-income countries in transplantation rates per million population (4). Among the solid organ Tx, kidneys are the most frequently transplanted organs. Other frequently transplanted organs include the liver, lungs, and heart (3). Despite advances in surgical techniques, immunosuppressant drug regimens, hospital care, and the identification methods of post-transplant complications, bacterial infections are still the most important causes of patients' mortality and morbidity following solid organ Tx (3, 5). Sites of infection vary in the literature, with the most common being urinary tract and surgical site infection (6). Urinary tract infection (UTI) is the most common complication after kidney transplantation, it affects 5%–36% of kidney transplant patients (KTPs) often associated with graft loss, increased healthcare cost, and mortality (7). UTI in KTPs is mainly due to underlying immunosuppression and the use of a urethral catheter with a greater predisposition to UTI (8).

The principal causes of UTIs in KTPs are similar to those observed in the general population with complicated UTIs (9). Gram-negative bacteria (GNB) cause up to 90% of UTIs after kidney transplantation. KTPs are particularly susceptible to infections caused by Enterobacteriaceae-producing extended-spectrum *β*-lactamases (ESBLs) and are of extreme clinical relevance (10). Approximately 10% of KTPs will develop a UTI caused by ESBLs within the first year, and these patients face a three times higher risk of recurrence compared to those infected with non-MDR pathogens (11). ESBLs are a large, rapidly evolving group of plasmid-enzymes that confer resistance to penicillins and first, second, and third-generation cephalosporins and aztreonam. ESBL-producing organisms may potentially develop resistance to other routinely used antibiotics for UTIs, such as fluoroquinolones, cotrimoxazole, or aminoglycosides challenging the management (12, 13). Insertion sequences, integrons, and transposons transferred between the microorganisms play a crucial role in the global dissemination of the ESBL genes (14).

Among the Enterobacteriaceae-producing ESBLs, *Klebsiella pneumoniae* is emerging as a prominent causative agent globally, particularly in cases of recurrent infections involving MDR strains, in KTPs. *K. pneumoniae* is a gram-negative, encapsulated, and non-motile bacteria (15). It is part of the normal flora and colonizes numerous human mucosal surfaces, including the distal urethra, upper respiratory tract, and gastrointestinal tract (15, 16). It is regarded as an opportunistic pathogen, frequently implicated in severe hospital-acquired infections, especially among immunocompromised patients and those with significant comorbidities (17). *K. pneumoniae* causes a wide range of infections, including pneumonia, UTIs, bacteremia, and liver abscesses (15, 17). The management of infections caused by ESBLs remains challenging, with limited antimicrobials available and scarce supporting evidence. Careful selection of empirical antibiotic therapy for UTIs is critical, and it should be guided by risk factors associated with infections (18).

Carbapenems have been considered as the front-line therapy both in the general population and in immunocompromised patients, including KTPs (19). Carbapenems, including imipenem and meropenem, are considered the drugs of choice for ESBL infections (20). These infections are usually resistant to penicillin, fluoroquinolones, trimethoprim–sulfamethoxazole, and some aminoglycosides (20). For carbapenem-resistant *K. pneumoniae* infection, prolonged infusion of carbapenems, particularly meropenem is a treatment option (21). Prolonged infusion maximizes the time that antibiotic concentrations remain above the minimum inhibitory concentration (MIC) of microorganisms, which reduces the chance of treatment failure (22). Here, we describe a multidrug-resistant *K. pneumoniae* isolated in renal transplant recipients with recurrent UTI treated with prolonged infusion of meropenem. The written consent was obtained from the patient to present this case.

## Case presentation

A 33-year-old male patient with a known history of hypertension and chronic kidney disease (CKD) secondary to chronic interstitial nephritis underwent first live-related renal transplantation surgery in 2015 (donor-mother). He was on triple immunosuppression (prednisolone + mycophenolate + tacrolimus) and had good allograft function with no incidence of UTI. However, 2.5 years post-transplant, he voluntarily discontinued his transplant clinic visits and stopped his immunosuppressants for which he had developed chronic allograft dysfunction, and he was initiated on hemodialysis after 5.5 years (duration 2.5 years; there was no catheter used during hemodialysis). He underwent a second kidney transplantation from a deceased donor (a donor 27-year-old male, with no diabetes/hypertension) in 2023. He received antithymocyte globulin (ATG) induction pre-transplantation and was on triple immunosuppression (prednisolone + mycophenolate + tacrolimus). He was discharged with serum creatinine of 0.98 mg/dl. The detailed treatment timeline is provided in Table 1.

**Table 1:** Treatment timeline table.

Date	Case presentation	Treatment
The case dates back to May 2015	It is a case of ESRD, He had B/L non-biopsiable kidneys at the time of presentation	HTN was initiated on hemodialysis and was on twice-weekly MHD
13th May 2015	✓ Live-related, ABO-compatible kidney transplantation with mother as donor ✓ Immediate graft function with good urine output ✓ Nadir serum creatinine was 0.7 mg/dl on POD 6	Started on triple immunosuppression with Tacrolimus, MMF, Steroids
The patient was discharged on POD 9 on triple immunosuppression. Serum creatinine at discharge: 0.8 mg/dl		
0–32 months post-transplant	✓ This period was uneventful ✓ The patient was maintaining serum creatinine: 0.7–1 mg/dl ✓ Tacrolimus level was maintained adequately	Triple immunosuppression with tacrolimus, MMF, steroids
32 months post-transplant	✓ He discontinued his transplant clinic visits for nearly 3.5 months ✓ He stopped his medications ✓ He presented with a serum creatinine level of 4.3 mg/dl ✓ Urine routine and USG transplant kidney doppler were normal ✓ Renal biopsy: acute cellular rejection category 1A (i2, t2). C4d staining was negative	✓ He was pulsed with IV Methylprednisolone (250 mg—three doses) ✓ He received ATG therapy
Next 3 Years	✓ Renal functions did not improve and remained elevated ✓ For the next 3 years his creatinine levels: 3.5–4.5 mg/dl	
5 ½ years post-transplant	✓ He started to develop uremic symptoms ✓ His serum creatinine worsened to 7.74 mg/dl ✓ He was registered for the deceased donor transplantation program	✓ He was initiated on hemodialysis via right IJC (Duration 2.5 years) ✓ His immunosuppression was tapered ✓ Since then, he has continued on twice-weekly maintenance hemodialysis
3 years later	✓ He underwent a deceased donor Kidney transplantation on 30/7/23 ✓ The patient had on table urine output of 60 ml/h ✓ Nadir Sr. creatinine was 0.95 mg/dl on POD 8	✓ 1.6 mg/kg ATG induction was given (cumulative dose) ✓ Was started on triple immunosuppression
The patient was discharged on POD 10, tacrolimus 3.5 mg/day, MMF 1.0 g/day, prednisolone 20 mg/day. Serum creatinine at discharge: 0.98 mg/dl		
10-days post-transplant	✓ He presented with high-grade fever with chills, pain, and swelling in the right side of the scrotum, dysuria ✓ On local examination of scrotal erythema, right testis tenderness ✓ USG scrotum and testis showed bilateral minimal hydrocele with internal septations on the right side and scrotal wall edema likely infected right hydrocele ✓ The patient was admitted; his serum creatinine levels were 1.45 and procalcitonin 0.72. Urine and blood cultures negative ✓ He was diagnosed with have infected right hydrocele	✓ He was treated with IV antibiotics, ceftriaxone 1 g BD, for 10 days and was continued on trimethoprim/sulfamethoxazole prophylaxis
He was discharged with the following immunosuppressive medications: tacrolimus, 3 mg/day; MMF, 1 g/day; steroids, 20 mg/day. His tacrolimus level was 6.63 ng/ml. At the time of discharge: creatinine, 1.08; TLC, 19,290		
22 days post-transplant	✓ The patient presented with high-grade fever with chills, pain, and swelling in the right scrotal region ✓ USG transplant Doppler showed raised cortical echoes of the left transplant kidney with echogenic sinuses; thickened and irregular urinary bladder wall; and pyelonephritis ✓ USG scrotum and testis showed right Epididymo-orchitis with right hydrocele ✓ His serum creatinine levels were 1.7, urine and blood culture were negative	✓ He was again treated for right epididymo-orchitis and UTI with IV antibiotics (meropenem) for 10 days
He was discharged with the following immunosuppressive medications: tacrolimus, 3 mg/day; MMF, 750 g/day; steroids, 17.5 mg/day. At the time of discharge: creatinine, 0.92; TLC, 13,990		
30-days post-transplant	✓ The patient was asymptomatic, but given elevated TLC, he underwent a USG transplant kidney Doppler ✓ USG transplant Doppler showed thickened and irregular urinary bladder and cystitis. Infected peri-graft collection measuring 23 ccs about the lower pole of the transplanted kidney ✓ USG-guided peri-graft fluid aspiration was done, and its culture showed no growth ✓ The patient continued to be asymptomatic despite elevated TLC levels	—
45-days post-transplant	✓ He presented with high-grade fever with chills and dysuria ✓ On examination, vitals are stable, systemic and local examination was normal, and no graft tenderness ✓ USG transplant Doppler showed raised cortical echoes of left transplant kidney with echogenic sinuses; thickened and irregular urinary bladder wall; pyelonephritis ✓ Urine microscopy yielded plenty of pus cells and urine culture yielded multidrug-resistant *Klebsiella pneumoniae* (10^5^ cfu/ml) ✓ AST Report is given in Table 2	✓ He was treated with meropenem 1 g over a 3-h infusion, colistin 3 million units loading dose followed by 1 million units TID, and nitrofurantoin 100 mg BD for 14 days
He was discharged with the following immunosuppressive medications: tacrolimus, 3 mg/day; MMF, 750 g/day; steroids, 15 mg/day. His tacrolimus level was 7.65 ng/ml. At the time of discharge: creatinine, 1.01; TLC, 10,050		
60-days post-transplant	✓ He again started to have persistent high-grade fever spikes ✓ Given recurrent epididymo-orchitis and UTI, urine was sent for AFB, TB PCR, KOH, and C/s to look for atypical organisms—all reports were negative	✓ He received 1 week's course of azithromycin for atypical coverage
70-days post-transplant	✓ He presented with high-grade fever with chills and dysuria ✓ On examination, he had hypotension ✓ Urine microscopy yielded plenty of pus cells and urine culture yielded multidrug-resistant *Klebsiella pneumoniae* (10^5^ cfu/ml) ✓ AST Report is given in Table 3	✓ He was treated with IV/oral antibiotics (meropenem, 1 g over a 3-h infusion, and doxycycline) for 10 days
He was discharged with the following immunosuppressive medications: tacrolimus, 3 mg/day; MMF, 500 g/day; steroids, 15 mg/day. At the time of discharge: creatinine, 1.38; TLC, 7,650		
85-days post-transplant	✓ He presented with high-grade fever with chills ✓ Given persistent fever episodes, he underwent a CT abdomen and CT thorax ✓ Normal study of thorax ✓ Bilateral chronic kidney disease of native kidneys ✓ Small and atrophic right transplant kidney in RIF ✓ Normal left transplant kidney in LIF ✓ Urine microscopy yielded plenty of pus cells and urine culture yielded multidrug-resistant *Klebsiella pneumoniae* (10^5^ cfu/ml) ✓ Repeat urine culture still showed the growth of multidrug-resistant *K. pneumoniae* ✓ AST report is given in Table 2 ✓ Given recurrent urinary tract infections, he underwent diagnostic cystoscopy on 28 October 2023 ✓ Intraoperative cystogram showed reflux of contrast into old right transplant kidney ✓ Despite 2 weeks of antibiotic therapy, the patient persisted in having high-grade fever spikes ✓ He underwent right subcapsular renal allograft nephroureterectomy and bilateral vassal ligation on 18/11/23	✓ He was started on meropenem 1 g over a 3-h infusion and colistin 3 million units loading dose followed by 1 million units TID for 14 days as per the culture report. ✓ Antibiotics were escalated to tigecycline (100 mg loading dose followed by 50 mg BD) and colistin (3 million units loading dose followed by 1 million units TID) for 14 days based on the culture and sensitivity pattern ✓ Postoperatively he was continued on IV antibiotics meropenem 2 g over 3-h infusion and colistin 3 million units loading dose followed by 1 million units for 14 days
Repeat urine c/s showed no growth, He was discharged with the following immunosuppressive medications: tacrolimus, 3 mg/day; MMF, 500 g/day; steroids, 10 mg/day. At the time of discharge: creatinine, 1.54; TLC, 11,950		
Events post-discharge to date	✓ The patient did not have any further episodes of fever in the last 5 months ✓ Repeat urine routine did not show any pus cells and repeat urine cultures were negative ✓ His serum creatinine: 1.6–1.7 mg/dl ✓ He completed 6 months of prophylaxis with valganciclovir and Bactrim ✓ Current immunosuppression: ○ Tacrolimus, 2 mg/day ○ MMF, 1 g/day ○ Steroids,10 mg/day ✓ Tac level maintained at 4–5 ng/ml	

ESRD, end-stage renal disease; HTN, hypertension; MHD, maintenance hemodialysis; POD, postoperative day; MMF, mycophenolate mofetil; USG, ultrasound sonography; IV, intravenous; ATG, antithymocyte globulin; IJC, internal jugular catheter; TLC, total leukocyte count; UTI, urinary tract infection; AFB, acid-fast bacilli; TB, tuberculosis; PCR, polymerase chain reaction.; KOH, potassium hydroxide; CFU, colony-forming units; CT, computed tomography; RIF, renal interstitial fibrosis.

Ten days post-transplant, he presented with swelling and pain in the right side of the scrotum, high-grade fever with chills, and episodes of burning micturition. On examination, vitals were stable, the systemic examination was normal, and there was no graft tenderness. Given pain in the right side of the scrotum, a urology opinion was taken, and ultrasound sonography (USG) scrotum and testis were performed which showed mild right infected hydrocele. He has been conservatively treated with IV antibiotics meropenem 1 g TID over a 3-h infusion for 10 days. He was continued on cotrimoxazole prophylactic dose during the hospital stay. Urine and blood culture showed no growth as he was already initiated on antibiotic treatment. He remained stable and was discharged with triple immunosuppressive medications (tacrolimus, mycophenolate mofetil, and steroids).

Twenty-two days post-transplant, he again presented with high-grade fever with chills, pain, and swelling in the right scrotal region. USG transplant Doppler showed raised cortical echoes of the left transplant kidney with echogenic sinuses and thickened and irregular urinary bladder wall. The urine routine showed plenty of inflammatory cells. He was being treated empirically with IV antibiotics meropenem 1 g TID over a 3-h infusion and nitrofurantoin 100 mg BD for 10 days. Urine culture showed no growth as he was already initiated on antibiotic treatment. After the antibiotic treatment, he was stable with no fever spikes and was discharged with triple immunosuppressive medications.

Thirty days post-transplant, he was asymptomatic, but given elevated total leukocyte count (TLC), he underwent USG transplant kidney Doppler. USG transplant Doppler showed thickened and irregular urinary bladder and peri-graft collection measuring 23 ccs about the lower pole of the transplanted kidney. USG-guided peri-graft fluid aspiration was done, and its culture showed no growth. He continued to be asymptomatic despite elevated TLC levels.

Forty-five days post-transplant, he presented with high-grade fever with chills and dysuria. On examination, his vitals were stable, systemic, and local examination was normal, and there was no graft tenderness. The urine routine showed plenty of inflammatory cells. He was empirically treated on IV antibiotics (meropenem 1 g over 3-h infusion, colistin 3 million units loading dose followed by 1 million units TID, and nitrofurantoin 100 mg BD for 14 days). Urine culture showed the growth of multidrug-resistant *Klebsiella pneumoniae*, blood culture showed no growth, and the same antibiotic treatment was continued after the culture report though it was MDR. After the antibiotic treatment, he was stable with no fever spikes and was discharged with immunosuppressive medications. The detailed antibiotic susceptibility pattern is provided in Table 2.

**Table 2:** AST report of MDR *Klebsiella pneumoniae.*

Antimicrobial agent	45 days post-transplant		85 days post-transplant	
	MIC	Interpretation	MIC	Interpretation
Amoxicillin and clavulanic acid	≥32	Resistant	≥32	Resistant
Piperacillin–tazobactam	≥128	Resistant	≥128	Resistant
Cefuroxime	≥64	Resistant	≥64	Resistant
Cefuroxime axetil	≥64	Resistant	≥64	Resistant
Ceftriaxone	≥64	Resistant	≥64	Resistant
Cefoperazone–sulbactam	≥64	Resistant	≥64	Resistant
Cefepime	≥32	Resistant	≥32	Resistant
Ertapenem	≥8	Resistant	≥8	Resistant
Imipenem	8	Resistant	8	Resistant
Meropenem	≥16	Resistant	≥16	Resistant
Amikacin	32	Resistant	32	Resistant
Gentamicin	≥16	Resistant	≥16	Resistant
Ciprofloxacin	≥4	Resistant	≥4	Resistant
Tigecycline	4	Intermediate	4	Intermediate
Fosfomycin	—	Resistant	—	Resistant
Trimethoprim–sulfamethoxazole	≥320	Resistant	≥320	Resistant
Colistin	≤0.5	Intermediate	≤0.5	Intermediate
Nitrofurantoin	—	Intermediate	—	Resistant

Sixty days post-transplant, he again started to have persistent high-grade fever spikes. Because of recurrent UTI, urine was sent for Ziehl–Neelsen staining, cartridge-based nucleic acid amplification test (CB-NAAT), KOH mount, and culture, but all reports were negative. On 70 days post-transplant, he presented with high-grade fever with chills and dysuria. On evaluation, he was found to have a UTI, because of which he was started on meropenem 1 g over a 3-h infusion TID for 10 days. Urine culture showed the growth of multidrug-resistant *Klebsiella pneumoniae*. Given recurrent UTI, a urology opinion was taken, and expressed prostatic secretions were sent for analysis—which showed no growth. After the antibiotic treatment, he was stable with no fever spikes and was discharged with immunosuppressive medications. The detailed antibiotic susceptibility pattern is provided in Table 3.

**Table 3:** AST report of MDR *Klebsiella pneumoniae* (70 days post-transplant).

Antimicrobial agent	MIC	Interpretation
Amoxicillin and clavulanic acid	≥32	Resistant
Piperacillin–tazobactam	≥128	Resistant
Cefuroxime	≥64	Resistant
Cefuroxime axetil	≥64	Resistant
Ceftriaxone	≥64	Resistant
Cefaperazone–sulbactam	≥64	Resistant
Cefepime	≥32	Resistant
Ertapenem	≥8	Resistant
Imipenem	8	Resistant
Meropenem	≥16	Resistant
Amikacin	32	Resistant
Gentamicin	≥16	Resistant
Ciprofloxacin	≥4	Resistant
Tigecycline	4	Intermediate
Fosfomycin	—	Resistant
Trimethoprim–sulfamethoxazole	≥320	Resistant
Colistin	≤0.5	Intermediate
Nitrofurantoin	—	Resistant
Cefoxitin	≥64	Resistant
Ceftizoxime	≥64	Resistant
Doxycycline	≥16	Resistant
Tetracycline	≥16	Resistant
Tobramycin	≥16	Resistant
Netilmicin	4	Sensitive
Ampicillin/sulbactam	≥32	Resistant
Ceftazidime–avibactam	≥16	Resistant
Chloramphenicol	≥64	Resistant
Ceftozolone/tazobactam	≥32	Resistant

Eighty-five days post-transplant, he presented with high-grade fever with chills. Because of persistent fever episodes and recurrent UTI, he underwent a CT abdomen which showed bilateral chronic kidney disease of native kidneys and a small atrophic right transplant kidney in renal interstitial fibrosis (RIF). Urine culture showed the growth of multidrug-resistant *K. pneumoniae* and urine routine showed plenty of inflammatory cells. He was started on meropenem 1 g over a 3-h infusion and colistin 3 million units loading dose followed by 1 million units TID for 14 days as per the culture report. However, he had subsequent fever spikes. Repeat urine culture still showed the growth of multidrug-resistant *K. pneumoniae*, and antibiotics were escalated to tigecycline (100 mg loading dose followed by 50 mg BD) and colistin (3 million units loading dose followed by 1 million units TID) for 14 days based on the culture and sensitivity pattern. Despite 2 weeks of antibiotic therapy, he persisted in having high-grade fever spikes, a urology opinion was taken, and he underwent a diagnostic cystoscopy with a cystogram which showed reflux of the contrast kidney in the old right transplant kidney. He underwent a transplant kidney subcapsular renal allograft nephroureterectomy and bilateral vassal ligation. Postoperatively he was continued on IV antibiotics meropenem 2 g over 3-h infusion and colistin 3 million units loading dose followed by 1 million units for 14 days. However, because of worsening renal functions, colistin was stopped, and only meropenem infusion was continued. He was afebrile post-procedure. Repeat urine culture showed no growth. He was discharged 6 days later with triple immunosuppressive medications. He did not have any further episodes of fever in the last 5 months. Repeat urine routine did not show any inflammatory cells, and repeat urine cultures showed no growth. He has completed 6 months of prophylaxis with valganciclovir and cotrimoxazole for 6 months and did not have further episodes of UTI. The detailed antibiotic susceptibility pattern is provided in Table 2.

## Discussion

The incidence of infections due to MDR gram-negative bacteria (GNB) in solid organ transplant recipients is on the rise. These patients are more prone to MDR GNB infections due to their prolonged hospital stay, as well as receiving immunosuppressive agents and broad-spectrum antibiotics. Infections caused by MDR GNB, particularly multidrug-resistant *Enterobacteriaceae* pose a therapeutic challenge due to limited therapeutic options.

The epidemiology of ESBL-producing and carbapenem-resistant bacteria is becoming more complex, and the limits between hospital and community settings are narrowing especially in the cases of KTPs (23). Such outpatients are frequently admitted to hospital. Thus, microorganism origin may not be obvious since patients could have acquired the strain during the last hospital admission, and just after discharge, a subsequent UTI can manifest. Delayed graft function, diabetes mellitus, previous antibiotic exposure, antibiotic prophylaxis, and relapsing UTI are independent risk factors for acquiring infections by ESBL-producing *E. coli* and *K. pneumoniae* (24). The high co-resistance to other antibiotics (non-*β*-lactams) found in ESBL-producing bacteria in UTI from KTPs remains a serious clinical challenge (25).

The most frequent antibiotics recommended for the treatment of MDR GNB include carbapenems (i.e., meropenem), colistin, fosfomycin, tigecycline, and aminoglycosides (26). Carbapenems, including imipenem and meropenem, are considered the drugs of choice for ESBL infections. Following intravenous infusion, carbapenems are distributed widely into various body fluids, which contributes to their efficacy for a wide range of infections (27). In terms of their pharmacodynamics profile, carbapenems display time-dependent bactericidal activity against gram-negative bacteria (27). Pharmacokinetic studies have shown that a prolonged infusion of 1 g meropenem for 3 h for every 8 h was able to retain the concentration above MIC (≤ 4 mg/dl) for a longer period in comparison with an infusion of a similar dose during half an hour (27, 28). Also, according to standard guidelines (IDSA 2023), this treatment protocol (1 g meropenem q8 h over 3 h) is more effective against carbapenamase-producing *K. pneumoniae*'s isolates with MIC ≤4 mg/dl, and for isolates with MIC >4 mg/dl, high-dose prolonged infusion (2 g meropenem q 8 h over 3 h) is recommended (29).

The decision to use a high dose of meropenem is based on the need to achieve adequate pharmacokinetic and pharmacodynamic target attainment and to possibly reduce the chances of the development of resistance (21). Indeed, extended infusion strategies have been associated with improved clinical response compared with bolus infusions of the same dose at higher MICs (27). Depending on the circulating strains and prior antibiotic treatment, some patients may be infected with carbapenem-resistant *K. pneumoniae* isolates that exhibit relatively low (<4 mg/L) or moderately elevated meropenem MICs (8–16 mg/L) (27).

In the present case, a renal transplant recipient with recurrent UTI infection with multidrug-resistant *K. pneumoniae* with meropenem MIC ≥16 mg/L was treated with extended infusion of meropenem (2 g TID infused over 3 h) and was associated with favorable outcomes. The clinical guidelines (as per IDSA 2023) indicate the use of high-dose meropenem (2 g TID infused over 3 h) for the treatment of multidrug-resistant pathogens and the duration of antibiotic treatment to 10–15 days in complicated UTI (29). The same dosage regimen was implemented. Though this patient was treated with colistin combined with meropenem and tigecycline colistin, the patient came back with recurrent UTI with MDR *K. pneumoniae*. This probably is because of a sub-optimal dose of colistin and tigecycline. The sub-optimal dose of colistin used was due to renal impairment. It could also be due to the PK/PD of tigecycline which does not reach a therapeutic concentration in the urine/kidney.

In a meta-analysis carried out by Lokhandwala et al. (30), to compare the treatment outcomes of patients who received meropenem through prolonged infusion vs. those who received it intermittently incorporated data from eight studies involving a total of 1,476 patients. They observed that prolonged meropenem infusion was associated with better outcomes. This included a reduced mortality rate and a higher success rate when compared to intermittent meropenem infusion. Similarly, a meta-analysis conducted by Chen et al. (31), which explored the comparison between continuous and intermittent meropenem infusion, reported that prolonged meropenem infusion was associated with better clinical outcomes.

Although these strains are reported as resistant to carbapenems by the microbiology laboratory, they are often treatable with higher-dose regimens in combination with other agents. Qureshi et al. (32) reported that in a cohort of 41 patients with carbapenem-resistant *K. pneumoniae* bacteremia, the highest clinical response rates were observed in patients who received a carbapenem-containing combination regimen, even though nearly one-third of the isolates were classified as carbapenem-resistant by current breakpoints.

These findings underscore the potential for substantial clinical improvement in treating multidrug-resistant infections through the adoption of prolonged meropenem infusion. In the present case, the patient was initially treated with meropenem 1 g over a 3-h infusion, but the patient came back with UTI recurrence. Also, with combination therapy, the patient came back with recurrent UTI. However, with a 2 g TID prolonged infusion of 3 h, the patient appears to have a longer duration of remission (5 months). We need to further follow up on the case to know how long the patient can remain in remission.

Kidney transplantation is a transformative medical intervention that significantly enhances the quality of life for patients. However, complications such as urinary tract infections (UTIs) can arise, sometimes due to underlying conditions like renal reflux. In this case, an intraoperative cystogram revealed reflux of contrast into an old right transplant kidney, suggesting it is the potential source of recurrent UTIs. The incidence of UTIs in renal transplant patients with vesicoureteral reflux is notably high, with some studies reporting rates as high as 80% and renal reflux rates of 2%–8% (33, 34).

Pretransplant nephrectomy is often recommended in patients with reflux to minimize infection risk. However, identifying renal reflux as a source of infection can be challenging because it is relatively uncommon and may only be suspected after excluding other infection sources. In this case, diagnostic cystoscopy played a critical role in identifying the cause of infection, which was resolved following nephrectomy. This highlights the importance of thorough diagnostic evaluations and tailored interventions in managing post-transplant complications (35, 36).

Following are the investigations to be carried out in suspected post-transplantation urinary tract infection (UTI) cases. Firstly, the baseline clinical observations to be documented like pyrexia and tachycardia. In per-abdominal examination, suprapubic and/or loin tenderness shall be noted. Genital, pelvic, and rectal examination may indicate an enlarged prostate gland, tender prostate, testis, and/or epididymis. In post-menopausal female cases, atrophic vaginitis may be noted. A urine dipstick analysis may show leukocyte esterase, nitrites, blood, and/or protein. If these results are suggestive of UTI, urine microscopy may reveal pyuria and organism, and urine culture results may reveal the etiological agent and resistance pattern of the organism isolated. Simultaneously, serum creatinine, C-reactive protein (CRP), fasting blood sugar, and HbA1C should be investigated. If there is the involvement of kidneys, serum creatinine and CRP (>100 mg/L is consistent with pyelonephritis) may be raised (37, 38).

To know the cause of recurrent UTI, the USG of the transplanted kidney, native kidney, and bladder has to be done. The post-micturition residual volume of urine should be noted down. In the USG, presence of stones, incomplete bladder emptying, and dilated pelvic–calyceal system should be noted. In the above investigations, if structural abnormalities are present, surgery or other corrective measures have to be taken. If no structural abnormality is noted on USG, voiding cystourethrography and urodynamic studies have to be performed. If vesicourethral reflux is present, it is corrected by surgery or endoscopic injection. If bladder dysfunction is present medical and/or surgical management is indicated. If blood sugar is high, it should be managed with oral and/or injectable anti-hyperglycemic agents (37, 38).

### Strengths

✓ The case report focuses on a recurrent UTI caused by multidrug-resistant *Klebsiella pneumoniae* in a renal transplant recipient. It addresses a critical clinical issue frequently encountered in transplant medicine, highlighting challenges associated with managing MDR infections in immunocompromised patients.
✓ The use of extended meropenem with higher dose for the treatment of MDR organisms is a noteworthy strategy. It highlights the potential benefits of this antibiotic administration technique, demonstrating its effectiveness against MDR pathogens.
✓ By presenting a successful outcome using an extended infusion of high-dose meropenem, the case contributes to the growing body of evidence supporting this approach, potentially guiding clinicians in similar in resource limited situations.
✓ This case report provides a comprehensive follow-up of the patient over several months, which enhances the reliability of the observations and supports the long-term efficacy of the treatment protocol.

### Limitations

✓ The findings may not be generalizable to a broader patient population, as this is a single case report. The results need validation through larger studies.
✓ Due to resource constraints, molecular tests could not be performed on the isolated multidrug-resistant (MDR) *Klebsiella pneumoniae* strains.

## Conclusion

Life-threatening infections caused by MDR and sometimes pan-drug-resistant gram-negative bacteria have increased dramatically in the last decade due to the irrational use of antimicrobial agents. This case and a few more cases described in the discussion suggest that prolonged high dose meropenem offers a more economically efficient therapeutic option for treating multidrug-resistant infections. Future randomized control trials, with larger and more diverse patient populations, should be conducted to corroborate our results and ascertain the optimal dosing and administration regimen for prolonged meropenem infusion. These studies will be instrumental in refining multidrug-resistant infection treatment protocols and ultimately optimizing patient care in the context of these life-threatening in resource limited settings.

## Data Availability

The original contributions presented in the study are included in the article/Supplementary Material; further inquiries can be directed to the corresponding author.
